# Prognostic impact of sarcopenia in patients with locally advanced adenocarcinoma of the esophagogastric junction treated with neoadjuvant chemoradiotherapy

**DOI:** 10.3389/fnut.2023.988632

**Published:** 2023-01-26

**Authors:** Jiao Ming, Rongxu Du, Jianhao Geng, Shuai Li, Zhiyan Liu, Yong Cai, Xianggao Zhu, Yangzi Zhang, Hongzhi Wang, Zhilong Wang, Lei Tang, Xiaotian Zhang, Zhi Peng, Aiwen Wu, Zhaode Bu, Yifan Peng, Yan Yan, Zhongwu Li, Yongheng Li, Ziyu Li, Weihu Wang

**Affiliations:** ^1^Key Laboratory of Carcinogenesis and Translational Research (Ministry of Education/Beijing), Department of Radiation Oncology, Peking University Cancer Hospital and Institute, Beijing, China; ^2^Key Laboratory of Carcinogenesis and Translational Research (Ministry of Education/Beijing), Department of Medical Imaging, Peking University Cancer Hospital and Institute, Beijing, China; ^3^Key Laboratory of Carcinogenesis and Translational Research (Ministry of Education/Beijing), Department of Gastrointestinal Oncology, Peking University Cancer Hospital and Institute, Beijing, China; ^4^Key Laboratory of Carcinogenesis and Translational Research (Ministry of Education/Beijing), Department of Gastrointestinal Surgery, Peking University Cancer Hospital and Institute, Beijing, China; ^5^Key Laboratory of Carcinogenesis and Translational Research (Ministry of Education/Beijing), Endoscopy Center, Peking University Cancer Hospital and Institute, Beijing, China; ^6^Key Laboratory of Carcinogenesis and Translational Research (Ministry of Education/Beijing), Department of Pathology, Peking University Cancer Hospital and Institute, Beijing, China

**Keywords:** sarcopenia, adenocarcinoma of the esophagogastric junction, neoadjuvant chemoradiotherapy, prognosis, nutritional indices

## Abstract

**Background:**

Few studies have evaluated the significance of sarcopenia in predicting the outcomes of patients with adenocarcinoma of the esophagogastric junction (AEG), especially those who received neoadjuvant chemoradiotherapy (NCRT). We aimed to identify the sarcopenic status and its impact on the outcomes of patients with locally advanced AEG who received NCRT followed by radical surgery or systemic therapy.

**Materials and methods:**

Patients with T3-4N+M0 AEG with accessible abdominal computed tomography (CT) before and after NCRT were retrospectively analyzed. Body composition parameters, particularly the skeletal muscle index (SMI), were assessed using a CT-based method, and sarcopenia was defined using a predetermined SMI cutoff value. Survival analysis was conducted using the Kaplan–Meier method. A Cox proportional hazards regression model was used to identify independent prognostic factors. Receiver operating characteristic curve analysis was carried out, and the area under the curve (AUC) was calculated to test the prognostic accuracy of different factors.

**Results:**

A total of 63 patients were enrolled, 65.1 and 79.4% of whom developed pre- and post-NCRT sarcopenia, respectively. Patients with pre-NCRT sarcopenia had lower radical surgery rates (70.7 *vs*. 95.5%, *p* = 0.047) than those without sarcopenia; however, sarcopenic status did not affect other short-term outcomes, including treatment-related toxicity and efficacy. Pre-NCRT sarcopenia was identified as an independent predictive factor for poor overall survival (OS) [adjusted hazard ratio (HR), 6.053; *p* = 0.002] and progression-free survival (PFS) (adjusted HR, 2.873; *p* = 0.031). Compared with nutritional indices such as the Nutritional Risk Screening 2002, weight loss during NCRT, and post-NCRT sarcopenia, pre-NCRT sarcopenia was regarded as the best predictive index for the 5-year OS (AUC = 0.735) and PFS rates (AUC = 0.770).

**Conclusion:**

Pre-NCRT sarcopenia may be an independent predictive factor for OS and PFS rates in patients with locally advanced AEG receiving multimodal treatment.

## 1. Introduction

The incidence of adenocarcinoma of the esophagogastric junction (AEG) has increased in Western and Asian countries in the past few decades (1, 2). AEG is highly aggressive, and most patients with this condition are at an advanced stage and have poor survival (3). Multimodal treatment, especially neoadjuvant chemoradiotherapy (NCRT) followed by surgery, has improved the overall survival (OS) of patients with AEG and is recommended as the standard treatment for locally advanced AEG (4). The results of our previous study also confirmed the efficacy of NCRT both in terms of downstaging and improving pathological response in patients with AEG (5).

Patients with locally advanced AEG typically present with progressive dysphagia, odynophagia, satiety, and unintentional weight loss (6), which usually leads to malnutrition. Most patients treated with neoadjuvant therapy (NAT) experience gastrointestinal toxicities such as anorexia, nausea, and emesis, which may aggravate malnutrition. Furthermore, malnutrition is considered a risk factor for adverse clinical outcomes in multiple tumors (7–9). In patients with gastric cancer and AEG, those who were at nutritional risk as assessed by the Nutritional Risk Screening 2002 (NRS 2002) and experienced weight loss, had more severe postoperative complications and poorer survival (7, 10, 11).

Recently, sarcopenia, defined as a loss of skeletal muscle mass and function, has been confirmed as a prognostic nutritional factor for poor outcomes in several types of cancer (12–15). In patients with upper gastrointestinal tract cancer who received NAT, emerging evidence has shown that sarcopenia affected NAT-related toxicity (16, 17), the clinical and pathological response to NAT (18, 19), postoperative complications (19, 20), and long-term survival (16). Sarcopenia is determined by skeletal muscle mass in body composition parameters which can be easily obtained from computed tomography (CT) images. As CT scan objectively demonstrates body composition and is performed routinely in patients with cancer, sarcopenia evaluated by CT method is an objective and reproducible nutritional parameter.

The significance of sarcopenia in predicting the outcomes of patients with AEG, especially those who received NCRT, has not been sufficiently evaluated in previous studies. Therefore, the aim of the present study was to identify the sarcopenic status before and after NCRT and its impact on severe treatment-related toxicity, efficacy of treatment, and survival outcomes in patients with locally advanced AEG who received NCRT.

## 2. Materials and methods

### 2.1. Patients

The study population included patients who received NCRT for locally advanced AEG at the Peking University Cancer Hospital between March 2011 and October 2017. The detailed inclusion criteria were as follows: (1) histologically proven AEG; (2) clinical diagnosis of T3-4N+M0 stage *via* endoscopic ultrasound or CT in accordance with the 8th edition American Joint Committee on Cancer (AJCC) Staging Manual; (3) chemoradiotherapy as the initial antitumor therapy; (4) a score of 0 or 1 in Eastern Cooperative Oncology Group performance status before treatment; and (5) accessible CT images of the abdomen before and after NCRT within 1 month or 1 to 2 months, respectively. Exclusion criteria were as follows: (1) combination with other malignant tumors; (2) incomplete clinical or pathological data; (3) no SOX or S-1 chemotherapy regimens during NCRT. Demographic, disease-related, and treatment information was obtained from the patients’ medical records.

All patients signed informed consent forms before antitumor therapy, including radiotherapy, chemotherapy, and surgery. The study was conducted in accordance with the Declaration of Helsinki and approved by the Ethics Committee of the Peking University Beijing Cancer Hospital and Institute (approval number: 2014KT74).

### 2.2. Treatment strategy

Details of the NCRT treatment strategy have been described in our previous study (5). In brief, all patients received RT with a total dose of 50 Gy to gross tumor and 45 Gy to high-risk lymphatic drainage area in 25 fractions along with concurrent SOX or S-1 chemotherapy. After completion of NCRT, patients without progression were candidates for radical surgery with total gastrectomy and D2 lymphadenectomy by experienced surgeons (21). Thereafter, postoperative adjuvant chemotherapy was considered based on the patient’s pathological results and physical tolerance. A multidisciplinary team provided accurate diagnoses and individualized therapy to patients who did not undergo radical surgery.

### 2.3. Short-term outcomes

The completion status of NCRT was recorded. NCRT-related toxicities were assessed weekly according to the Common Terminology Criteria for Adverse Events version 4.0, in which severe toxicity was defined as more severe than two grades. Within 1 to 2 months of the completion of NCRT, patients were evaluated for CT-based clinical response in accordance with the RECIST 1.1 criteria (22). The radical surgery rate was calculated. For patients who received surgery, the D2 lymphadenectomy rate, R0 resection rate, and severe complications of surgery, were collected. The pathological response evaluation was recorded using the tumor regression grade (TRG) per the NCCN guidelines (23).

### 2.4. Follow-up and long-term outcomes

All patients were followed up every 3 months for the first 2 years after the completion of treatment, every 6 months for the next 3 years, and annually thereafter. Follow-up evaluation included medical history, physical examination, cancer biomarker blood tests, thoracic X-rays or CT, and abdominopelvic CT or ultrasonography.

Overall survival (OS) was defined as the time from the date of histological diagnosis to the last date of follow-up or death, whereas progression-free survival (PFS) was defined as the time from the date of histological diagnosis to the date of the progression at primary tumor or metastatic lymph nodes after NCRT, any relapse at local or regional sites after radical surgery, new distant metastasis, the last date of follow-up, or death.

### 2.5. Assessment of body composition parameters and other nutritional indices

**FIGURE 1:**
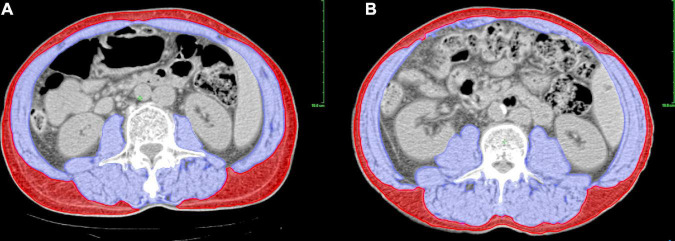
Assessment of body composition parameters using a CT-based method at L3 level. The picture shows different sarcopenic statuses in patients with the same BMI, in which blue and red zones represent the skeletal muscle and subcutaneous adipose tissue, respectively. **(A)** A male patient with sarcopenia (SMI = 46.52 cm^2^/m^2^, BMI = 22.50 kg/m^2^). **(B)** A male patient without sarcopenia (SMI = 56.19 cm^2^/m^2^, BMI = 22.50 kg/m^2^). BMI, body mass index; CT, computed tomography; L3, third lumbar vertebra; SMI, skeletal muscle index.

Unenhanced CT images of the abdomen before and after NCRT within 1 month and 1 to 2 months, respectively, were retrieved for analysis. A single CT image of the third lumbar vertebra (L3) with visible transverse and spinous processes was used to measure the cross-sectional area of skeletal muscle and subcutaneous adipose tissue (24) using Varian’s Eclipse software (version 15.6), as shown in Figure 1. The specific CT Hounsfield unit (HU) range used to identify and demarcate skeletal muscle was −29 to +150 and that for subcutaneous adipose tissue was −190 to −30 (25). The boundaries of the structures were manually corrected if necessary. Body composition parameters were assessed by one investigator (S Li) who was blinded to the patients’ information to eliminate measurement bias. The cross-sectional area (cm^2^) and radiation attenuation (HU) of the structures were then obtained. The muscle and tissue areas were normalized to the patient’s height to calculate the skeletal muscle index (SMI, cm^2^/m^2^) and subcutaneous adipose tissue index (SATI, cm^2^/m^2^). Sarcopenia was defined as SMI < 52.4 cm^2^/m^2^ for men and < 38.5 cm^2^/m^2^ for women, based on the cutoff value used by Prado et al. (26), which has been proven applicable to AEG (16, 27). The mean HU for skeletal muscle was defined as skeletal muscle density (SMD).

Two other nutritional indices, the NRS 2002 score and weight loss during NCRT, were also evaluated in this study. NRS 2002 is a nutritional screening tool, proposed by the European Society for Clinical Nutrition and Metabolism, to investigate the status of nutrition risk for patients in hospital. It consists of three parts: (1) nutritional status (score 0–3 points), which is evaluated according to the indicators of weight loss, food intake, and body mass index (BMI); (2) severity of disease (score 0–3 points); (3) age (score 0–1 points). The above three parts are added together to get the total score (0–7 points), in which patients scoring 3 or more are at risk nutritionally (28). All patients received the evaluation of NRS 2002 by a trained nurse at initial diagnosis. We also collected patients’ weight before and after NCRT in order to calculate the percentage of weight change during NCRT.

### 2.6. Statistical analysis

The normality of continuous data was determined using the Shapiro-Wilk test. Normally distributed continuous variables are presented as mean ± standard deviation (SD) and were compared using independent-samples or paired *t*-tests. Variables with a skewed distribution are presented as median (interquartile range) and were compared using the Mann–Whitney *U* test or Wilcoxon signed-rank test. Categorical data are presented as numbers and percentages and were compared using the *χ^2^* test, Fisher’s exact test, or Mann–Whitney *U* test. X-tile program was used to determine the optimal cutoff value of weight loss for predicting OS by selecting the highest *χ^2^* value (version 3.6.1; Yale University) (29). Survival analysis was conducted using the Kaplan–Meier method, and the results were compared using the log-rank test. Univariate and multivariate survival analyses were performed using Cox proportional hazards regression models. Clinical, pathological, and nutritional factors (age, gender, differentiation, Lauren type, cTNM stage, concurrent chemotherapy regimen, NRS 2002 score, weight loss during NCRT, pre- and post-sarcopenia, and radical surgery) that may influence the survival outcomes were included in the univariate analysis. All variables with *p* values < 0.10 in the univariate analysis were included in the multivariate analysis. Statistical significance was set at *p* < 0.05. Data were analyzed using IBM SPSS software (version 25.0; Chicago, IL, USA). The prognostic accuracy of factors was tested using the receiver operating characteristic (ROC) curve and compared with the area under the curve (AUC) value using R software (version 4.1.2).

## 3. Results

### 3.1. Clinicopathological characteristics and body composition parameters

A total of 63 patients with locally advanced AEG who received NCRT were enrolled in our study. Among them, 41 (65.1%) and 50 (79.4%) had pre- and post-NCRT sarcopenia, respectively, showing an increased incidence of sarcopenia during NCRT (χ^2^ = 20.661, *p* < 0.001). The clinicopathological characteristics classified by pre- and post-NCRT sarcopenia status are summarized in Table 1. Our data showed that compared to patients without sarcopenia, patients with pre-NCRT sarcopenia were associated with older age (64.4 ± 5.5 *vs*. 59.9 ± 6.5 years, *p* = 0.005) and had a lower proportion of Lauren intestinal type (48.8 *vs*. 77.3%, *p* = 0.029). The other clinicopathological characteristics did not differ significantly between patients with and without sarcopenia, both pre- and post-NCRT.

**TABLE 1:** Clinicopathological characteristics of patients according to pre- and post-NCRT sarcopenic status (*n* = 63).

Characteristics		Pre-NCRT			Post-NCRT		
	**Total (*n* = 63)**	**Sarcopenia (*n* = 41)**	**Non-sarcopenia (*n* = 22)**	***P*-value**	**Sarcopenia (*n* = 50)**	**Non-sarcopenia (*n* = 13)**	***P*-value**
Age (years), mean ± SD	62.8 ± 6.2	64.4 ± 5.5	59.9 ± 6.5	**0**.**005**§*	63.5 ± 6.3	60.2 ± 5.1	0.084§
Male gender, n (%)	60 (95.2)	38 (95.1)	21 (95.5)	>0.999	48 (96.0)	12 (92.3)	0.506‡
ECOG, n (%)				0.979			0.888
0	50 (79.4)	32 (78.0)	18 (81.8)		39 (78.0)	11 (84.6)	
1	13 (20.6)	9 (22.0)	4 (18.2)		11 (22.0)	2 (15.4)	
NRS 2002 score, n (%)				>0.999			0.631
<3	53 (84.1)	34 (82.9)	19 (86.4)		41 (82.0)	12 (92.3)	
≥3	10 (15.9)	7 (17.1)	3 (13.6)		9 (18.0)	1 (7.7)	
Differentiation, n (%)				0.493			0.592
Well to moderately	25 (39.7)	15 (36.6)	10 (45.5)		19 (38.0)	6 (46.2)	
Poorly	38 (60.3)	26 (63.4)	12 (54.5)		31 (62.0)	7 (53.8)	
Siewert type, n (%)				0.598†			0.771†
Siewert II	37 (58.7)	23 (56.1)	14 (63.6)		30 (60.0)	7 (53.8)	
Siewert III	20 (31.7)	14 (34.1)	6 (27.3)		15 (30.0)	5 (38.5)	
Unavailable	6 (9.5)	4 (9.8)	2 (9.1)		5 (10.0)	1 (7.7)	
Lauren type				**0**.**041**†*			0.279†
Intestinal type	37 (58.7)	20 (48.8)	17 (77.3)		27 (54.0)	10 (76.9)	
Diffuse type	11 (17.5)	9 (22.0)	2 (9.1)		11 (22.0)	0 (0.0)	
Mixed type	11 (17.5)	9 (22.0)	2 (9.1)		9 (18.0)	2 (15.4)	
Unavailable	4 (6.3)	3 (7.3)	1 (4.5)		3 (6.0)	1 (7.7)	
Clinical T category, n (%)				>0.999			>0.999
T3	14 (22.2)	9 (22.0)	5 (22.7)		11 (22.0)	3 (23.1)	
T4	49 (77.8)	32 (78.0)	17 (77.3)		39 (78.0)	10 (76.9)	
Clinical N category, n (%)				0.106†			0.078†
N1	18 (28.6)	9 (22.0)	9 (40.9)		12 (24.0)	6 (46.2)	
N2	32 (50.8)	22 (53.7)	10 (45.5)		26 (52.0)	6 (46.2)	
N3	13 (20.6)	10 (24.4)	3 (13.6)		12 (24.0)	1 (7.7)	
Clinical TNM stage, n (%)				0.538‡			>0.999‡
III	61 (96.8)	39 (95.1)	22 (100.0)		48 (96.0)	13 (100.0)	
IVA	2 (3.2)	2 (4.9)	0 (0)		2 (4.0)	0 (0)	
Concurrent chT, n (%)				0.116			0.566
S-1	16 (25.4)	13 (31.7)	3 (13.6)		14 (28.0)	2 (15.4)	
SOX	47 (74.6)	28 (68.3)	19 (86.4)		36 (72.0)	11 (84.6)	

chT, chemotherapy; ECOG, Eastern Cooperative Oncology Group; NCRT, neoadjuvant chemoradiotherapy; NRS, Nutritional Risk Screening; SD, standard deviation; SOX, S-1 and oxaliplatin.

*Statistically significant values are given in bold.

^†^Mann-Whitney *U* test.

^‡^Fisher’s exact test.

^§^*t*-test. χ^2^ test was used unless otherwise specified.

In addition, we investigated the relationship between the body composition parameters and sarcopenia. The results indicated that patients with pre-NCRT sarcopenia had lower weight (66.9 ± 10.9 *vs*. 77.2 ± 11.6 kg, *p* = 0.001) and SATI values (34.6 ± 18.1 *vs*. 52.2 ± 21.9 cm^2^/m^2^, *p* = 0.001) than those of non-sarcopenia patients. Post-NCRT sarcopenia patients also tended to have lower weight (65.6 ± 10.6 *vs*. 78.7 ± 9.8 kg, *p* < 0.001), SATI values (31.9 ± 14.6 *vs*. 53.8 ± 18.4 cm^2^/m^2^, *p* < 0.001), and SMD values (35.9 ± 6.7 *vs*. 40.0 ± 6.2 HU, *p* = 0.048) than those without sarcopenia.

### 3.2. Short-term outcomes

Among the 63 patients, 48 (76.2%) completed NCRT as expected. The vast majority (56, 88.9%) experienced benefits due to NCRT in the clinical response evaluation; among them, six patients did not undergo surgery (two were unsuitable for surgery and four refused surgery). In addition, seven patients had disease progression and received systemic therapy instead. Ultimately, 50 (79.4%) patients underwent radical surgery. Patients with pre-NCRT sarcopenia had lower radical surgery rates (70.7 *vs*. 95.5%, *p* = 0.047) than those without sarcopenia. However, completion status, severe toxicity, and clinical response distribution associated with NCRT did not differ significantly between patients with and without sarcopenia. There were also no significant differences in surgical outcomes in terms of D2 lymphadenectomy rate, R0 resection rate, severe complications, and the pathological response evaluation among patients with different sarcopenic statuses (Supplementary Table 1).

### 3.3. Long-term outcomes

**FIGURE 2:**
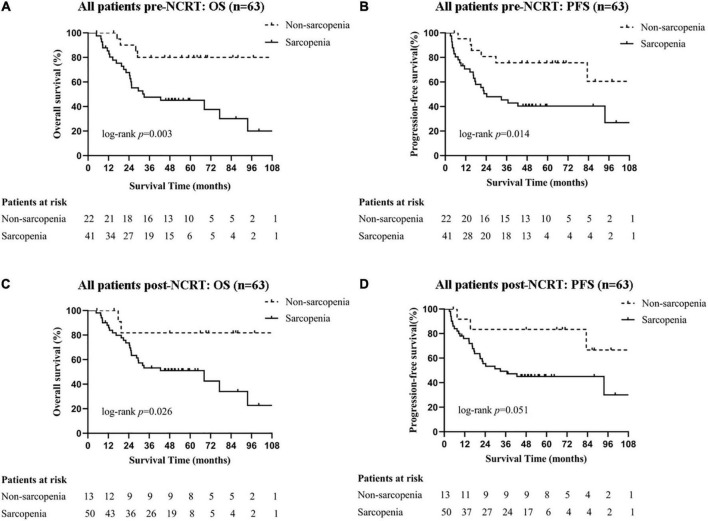
Survival curves of all patients with different sarcopenic statuses. Pre-NCRT **(A)** OS and **(B)** PFS. Post-NCRT **(C)** OS, and **(D)** PFS. NCRT, neoadjuvant chemoradiotherapy; OS, overall survival; PFS, progression-free survival.

The median follow-up time was 59.6 months (95% CI 54.1–65.1) for all patients. As shown in Figure 2, patients with pre-NCRT sarcopenia had significantly poorer OS rates among all patients: the 1-, 3-, and 5-year OS rates for patients with pre-NCRT sarcopenia were 85.3, 47.7, and 45.2%, respectively, whereas those for patients without sarcopenia were 100, 80.0, and 80.0%, respectively (*p* = 0.003). Additionally, patients with pre-NCRT sarcopenia had poor PFS rates: the 1-, 3-, and 5-year PFS rates for patients with pre-NCRT sarcopenia were 70.6, 45.4, and 40.0%, respectively, whereas those for patients without sarcopenia were 95.2, 75.6, and 75.6%, respectively (*p* = 0.014). Patients with post-NCRT sarcopenia had a significantly poorer OS rate (*p* = 0.026) and tended to have a poorer PFS rate (*p* = 0.051) than those without sarcopenia.

### 3.4. Prognostic accuracy of nutritional indices

**FIGURE 3:**
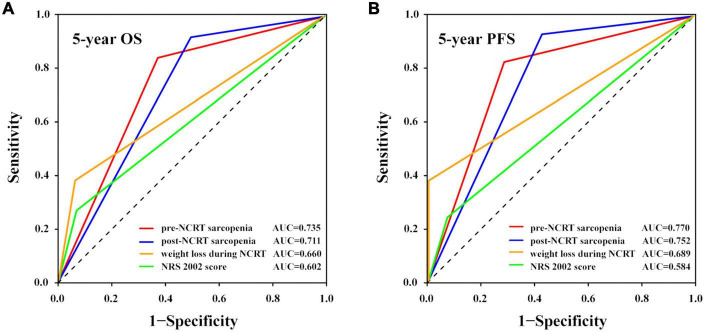
Prognostic accuracy of different nutritional indices compared using ROC curves with AUC values. The **(A)** 5-year OS and **(B)** 5-year PFS rates. AUC, area under the curve; NCRT, neoadjuvant chemoradiotherapy; NRS 2002, nutritional risk screening 2002; OS, overall survival; PFS, progression-free survival; ROC, receiver operating characteristic.

As sarcopenia was proven to be a significant predictive factor for survival, we tried to identify whether other nutritional indices, including the NRS 2002 score and weight loss during NCRT, could also predict the prognosis and, if so, their accuracy. The cutoff value for weight loss was 8%, calculated using the X-tile program, and the patients were classified into two groups according to the cutoff value. Patients with NRS 2002 scores of ≥ 3 had significantly lower OS (*p* = 0.008) and PFS rates (*p* = 0.029) than those with NRS 2002 scores of < 3. Patients with ≥ 8% weight loss during NCRT had significantly lower OS (*p* = 0.003) and PFS rates (*p* = 0.012) than those who did not meet this criterion. We then tested the prognostic accuracy of these indices using AUC models (Figure 3). The results indicated that pre-NCRT sarcopenia was the best predictive index for 5-year OS and PFS rate, with AUC values of 0.735 and 0.770, respectively.

### 3.5. Univariate and multivariate analyses for OS and PFS

**FIGURE 4:**
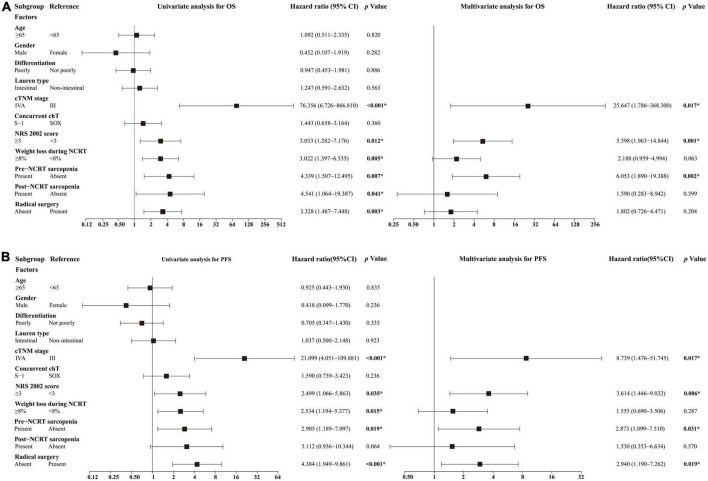
Results of univariate and multivariate analyses for **(A)** OS and **(B)** PFS. *Statistically significant values are given in bold. chT, chemotherapy; NCRT, neoadjuvant chemoradiotherapy; OS, overall survival; PFS, progression-free survival; SOX, S-1 and oxaliplatin.

We performed univariate and multivariate analyses in all patients who received NCRT to identify the independent predictive factors for OS and PFS (Figure 4). Univariate analysis showed that cTNM stage, NRS 2002 score, weight loss during NCRT, pre- and post-NCRT sarcopenia, and radical surgery were predictive factors of OS and PFS. Among these factors, multivariate analysis further identified that cTNM stage IVA [hazard ratio (HR) 25.647, 95% CI 1.786–368.300, *p* = 0.017], NRS 2002 score ≥ 3 (HR 5.398, 95% CI 1.963–14.844, *p* = 0.001), and pre-NCRT sarcopenia (HR 6.053, 95% CI 1.890–19.388, *p* = 0.002) were independent predictive factors for poor OS. The cTNM IVA stage (HR 8.739, 95% CI 1.476–51.745, *p* = 0.017), NRS 2002 score ≥ 3 (HR 3.614, 95% CI 1.446–9.032, *p* = 0.006), pre-NCRT sarcopenia (HR 2.873, 95% CI 1.099–7.510, *p* = 0.031), and absence of radical surgery (HR 2.940, 95% CI 1.190–7.262, *p* = 0.019) were proven to be independent predictive factors for poor PFS.

## 4. Discussion

In this study, we assessed sarcopenic status using a CT-based method before and after NCRT and determined the significance of sarcopenia in predicting poor OS and PFS rates in patients with locally advanced AEG. We also tested the prognostic accuracy of different nutritional indices to predict survival, of which pre-NCRT sarcopenia was the best predictive factor for 5-year OS and PFS rates. To the best of our knowledge, this is the first study to investigate the sarcopenic status before and after NCRT and identify its impact on outcomes in patients with multimodally treated AEG.

The impact of sarcopenia on poor survival has been determined in multiple cancers (12–15). However, there is a paucity of literature on patients with AEG, and inconsistent results have been obtained in some studies that involved patients with AEG (16, 17, 27). In the present study, pre-NCRT sarcopenia was demonstrated to be an independent predictive factor for OS (*p* = 0.002) and PFS (*p* = 0.031) in multivariate analysis, which may be related to the low proportion of the Lauren intestinal type (*p* = 0.029) and low radical surgery rate (*p* = 0.047). Studies have identified that the intestinal type is associated with favorable prognosis in gastric and AEG patients (30); patients who received NCRT plus radical surgery also had better prognoses than those who did not undergo radical surgery. However, considering the complexity of surgical decision making, which is not only related to complete resectability but also the patient’s physical condition including tolerance to surgery and willingness to undergo surgery, the results should be interpreted cautiously. In line with our study, Tan et al. also reported the prognostic impact of sarcopenia on poor OS in patients with esophagogastric cancer who underwent NAT and radical surgery (median OS: sarcopenia, 569 days *vs*. non-sarcopenia, 1,013 days; *p* = 0.04), in which 18% of patients had gastroesophageal junction cancers (16). Järvinen et al. also identified that patients with a reduction in SMI during NAT had poor survival (27). Conversely, other studies found no correlation between sarcopenia and survival outcome (17).

In addition, our study demonstrated that pre-NCRT sarcopenia had prognostic superiority in predicting the 5-year OS and PFS rates in patients with locally advanced AEG compared to NRS 2002 score and weight loss. The assessment of sarcopenic status using CT-based methods is objective, quantitative, timely, repeatable, non-invasive and does not require additional medical resources as CT examinations are performed routinely at initial diagnosis. Therefore, we can routinely assess sarcopenic status in all AEG patients receiving multimodal therapy. In comparison, NRS 2002 is a quick and convenient tool to perform initial screening for nutritional risk in hospitalized patients, and previous research has shown that it has prognostic value in postoperative complications and survival in esophageal, gastric, and other cancers (7–9). However, as a rapid nutritional screening method, NRS 2002 depends on patients’ self-reported value of weight loss and food intake, and it is only the first step in determining patients’ nutritional status (28). Therefore, its accuracy as a prognostic indicator may be limited. To further determine the nutritional status of patients, a comprehensive and detailed nutritional assessment is required. As for weight loss, it has been found that excessive weight loss during NAT or after surgery is associated with severe postoperative complications and worse survival in multiple cancers (10, 11, 31). Nevertheless, weight is affected by many factors. For example, weight gain can result from malignant pleural effusion or ascites due to tumor progression or hypoproteinemia, and weight loss can be caused by dehydration due to acute diarrhea. Therefore, weight loss may not accurately reflect the nutritional status of patients. In addition, only through weight monitoring over a period of time can weight loss be determined, so the nutritional status of patients cannot be assessed timely through weight loss.

In contrast to the generally accepted notion of poor survival in sarcopenic patients, limited studies have reported inconsistent conclusions on the short-term outcomes in patients receiving NAT (16–20, 32, 33). As for the NAT-related toxicity, Panje et al. observed an increased percentage of grade ≥ 3 toxicities during NCRT in pre-NCRT sarcopenic patients (83.3 *vs*. 52.4%, *p* = 0.04) (17), while our study and other studies revealed negative results (32). We also observed no effect of sarcopenia on clinical or pathological responses to NAT, consistent with several previous studies (20, 34). However, others studies found that sarcopenic patients had lower clinical and pathological response rates (18, 19). In addition, neither previous studies (17, 32, 33) nor our study could demonstrate a relationship between sarcopenia and postoperative complications. Although some other studies reported that post-NAT sarcopenia was associated with an increased occurrence of postoperative complications, especially pneumonia (19, 20). Further research is needed to clarify the role of sarcopenia in predicting toxicity and efficacy of multimodal treatment in patients with AEG.

The relationship between clinicopathological characteristics, body composition parameters, and sarcopenia in AEG patients was also investigated in our study. Our cohort was predominantly male (95.2%), with a mean age of 62.8 years, which is consistent with data from a larger group in a Chinese study (35). Post-NCRT sarcopenia was related to reduced SMD (*p* = 0.048), suggesting that NCRT may cause a reduction in the quality of skeletal muscles (34). Sarcopenia is associated with aging and usually occurs in older individuals (11). Our results also showed that pre-NCRT sarcopenia was associated with older age (*p* = 0.005), consistent with the results of other studies (20, 27, 33). Moreover, along with other studies, we found associations between sarcopenia and lower weight both before and after NCRT (27).

The underlying mechanisms by which sarcopenia develops and influences survival in patients with cancer remain obscure. Various candidate mechanisms, driven by multiple factors related to metabolism and inflammation, have been described (36). First, imbalances in protein metabolism lead to the overall loss of skeletal muscle and development of sarcopenia (36, 37). Imbalances in protein metabolism are associated with malnutrition which is a risk factor for poor survival in cancer patients (7). Whether nutritional supplementation could improve sarcopenic status and improve prognoses, however, requires further investigation. Second, proinflammatory mediators such as tumor necrosis factor-α and interleukin-6 play a pivotal role in the development and progression of sarcopenia (38). Some studies have shown that systemic inflammation is related to poor survival in several cancers (39, 40), and it is also among the most prominent features of sarcopenia (41). Third, cancer treatment, particularly chemotherapy, can cause direct damage to muscle tissue *via* molecular pathways (36). Additionally, other factors related to aging, such as reduced physical activity and a decline in anabolic hormones, may lead to sarcopenia (36). Further studies are required to confirm the hypotheses.

The current study has some limitations. First, owing to the retrospective nature of this study, many patients were excluded due to the lack of available CT images, which might have caused selection bias. In addition, differences in the timing of CT examinations among patients may contribute to the reduced prediction accuracy of sarcopenia on survival. Next, the study’s sample size was relatively small, and it was conducted in a single institution, which might have confined its external validity and affected the results, especially for short-term outcomes. Lastly, given few patients had changes in sarcopenia status before and after NCRT, we were not able to assess the impact of changes in sarcopenia status (i.e., patients with sarcopenia before NCRT but without after NCRT and vice versa) on outcomes, although it would be meaningful to conduct such studies. Thus, further prospective studies involving larger sample sizes and multiple institutions are required to confirm our results.

## 5. Conclusion

Pre-NCRT sarcopenia may be an independent predictive factor for poor OS and PFS rates in patients with locally advanced AEG treated with NCRT and had prognostic superiority in predicting the 5-year OS and PFS rates compared with other nutritional indices. Our findings imply that early screening for sarcopenic status and timely nutritional intervention for patients with sarcopenia may improve their survival outcomes.

## Data availability statement

The original contributions presented in this study are included in the article/Supplementary material, further inquiries can be directed to the corresponding authors.

## Ethics statement

The studies involving human participants were reviewed and approved by the Ethics Committee of the Peking University Beijing Cancer Hospital and Institute (approval number: 2014KT74). The patients/participants provided their written informed consent to participate in this study.

## Author contributions

WW, ZiL, and YL contributed equally in designing and supervising this study. JM analyzed the data and wrote this manuscript. RD and JG made contributions in collecting the data. SL assessed the body composition parameters in CT images. XgZ, YZ, and HW assisted in data interpretation and manuscript revision. ZyL, YC, XtZ, ZP, AW, ZB, and YP provided clinical data and contributed to the patient management. ZW and LT provided medical imaging data and diagnosis. YY and ZwL provided endoscopic data, pathological data, and diagnosis. All authors read and approved the final manuscript.
