# Supplementary material for: Prognostic impact of sarcopenia in patients with locally advanced adenocarcinoma of the esophagogastric junction treated with neoadjuvant chemoradiotherapy
Source: Front Nutr. 2023 Jan 26;10:988632. doi: 10.3389/fnut.2023.988632 (PMC9909020; doi:10.3389/fnut.2023.988632)
Supplement: Supplementary file 1 [file Table_1.docx]

**Supplementary Table 1. Short-term outcomes of patients according to pre- and post-NCRT sarcopenic status (n=63).**

| Variables, n (%) |  |  | pre-NCRT | | |  | post-NCRT | | |
| --- | --- | --- | --- | --- | --- | --- | --- | --- | --- |
|  | Total  (n=63) |  | Sarcopenia  (n = 41) | Non-sarcopenia  (n = 22) | *p*-value |  | sarcopenia  (n = 50) | non-sarcopenia  (n = 13) | *p*-value |
| Completion status of NCRT |  |  |  |  | 0.087 |  |  |  | 0.305 |
| as expected | 48 (76.2) |  | 34 (82.9) | 14 (63.6) |  |  | 40 (80.0) | 8 (61.5) |  |
| had adjustment | 15 (23.8) |  | 7 (17.1) | 8 (36.4) |  |  | 10 (20.0) | 5 (38.5) |  |
| Severe toxicity of NCRT | 18 (28.6) |  | 11 (26.8) | 7 (31.8) | 0.676 |  | 14 (28.0) | 4 (30.8) | ＞0.999 |
| Clinical response of NCRT |  |  |  |  | 0.162^†^ |  |  |  | 0.078^†^ |
| CR+PR | 27 (42.9) |  | 16 (39.0) | 11 (50.0) |  |  | 19 (38.0) | 8 (61.5) |  |
| SD | 29 (46.0) |  | 18 (43.9) | 11 (50.0) |  |  | 24 (48.0) | 5 (38.5) |  |
| PD | 7 (11.1) |  | 7 (17.1) | 0 (0) |  |  | 7 (14.0) | 0 (0) |  |
| Receive radical surgery | 50 (79.4) |  | 29 (70.7) | 21 (95.5) | **0.047*** |  | 38 (76.0) | 12 (92.3) | 0.363 |
| Variables, n (%) |  |  | pre-NCRT | | |  | post-NCRT | | |
|  | Surgical  (n=50) |  | Sarcopenia  (n = 29) | Non-sarcopenia  (n = 21) | *p*-value |  | sarcopenia  (n = 38) | non-sarcopenia  (n = 12) | *p*-value |
| D2 Lymphadenectomy | 48 (96.0) |  | 29 (100.0) | 19 (90.5) | 0.171^‡^ |  | 38 (100.0) | 10 (83.3) | 0.054^‡^ |
| R0 resection | 49 (98.0) |  | 28 (96.6) | 21 (100) | ＞0.999^‡^ |  | 37 (97.4) | 12 (100.0) | ＞0.999^‡^ |
| ypT category |  |  |  |  | 0.890 |  |  |  | 0.631 |
| T0-2 | 22 (44.0) |  | 13 (44.8) | 9 (42.0) |  |  | 16 (42.1) | 6 (50.0) |  |
| T3-4 | 28 (56.0) |  | 16 (55.2) | 12 (57.1) |  |  | 22 (57.9) | 6 (50.0) |  |
| ypN category |  |  |  |  | 0.851 |  |  |  | 0.515 |
| N0 | 35 (70.0) |  | 20 (69.0) | 15 (71.4) |  |  | 28 (73.7) | 7 (58.3) |  |
| N1-2 | 15 (30.0) |  | 9 (31.0) | 6 (28.6) |  |  | 10 (26.3) | 5 (41.7) |  |
| ypTNN stage |  |  |  |  | 0.213 |  |  |  | 0.560^‡^ |
| 0-II | 46 (92.0) |  | 25 (86.2) | 21 (100.0) |  |  | 34 (89.5) | 12 (100.0) |  |
| III | 4 (8.0) |  | 4 (13.8) | 0 (0) |  |  | 4 (10.5) | 0 (0.0) |  |
| TRG |  |  |  |  | 0.253^†^ |  |  |  | 0.212^†^ |
| 0 | 7 (14.0) |  | 5 (17.2) | 2 (9.5) |  |  | 4 (10.5) | 3 (25.0) |  |
| 1 | 16 (32.0) |  | 10 (34.5) | 6 (28.6) |  |  | 11 (28.9) | 5 (41.7) |  |
| 2 | 22 (44.0) |  | 12 (41.4) | 10 (47.6) |  |  | 20 (52.6) | 2 (16.7) |  |
| 3 | 5 (10.0) |  | 2 (6.9) | 3 (14.3) |  |  | 3 (7.9) | 2 (16.7) |  |
| Severe complications of surgery |  |  |  |  |  |  |  |  |  |
| Overall | 18 (36.0) |  | 9 (31.0) | 9 (42.9) | 0.390 |  | 13 (34.2) | 5 (41.7) | 0.901 |
| Pneumonitis | 5 (10.0) |  | 2 (6.9) | 3 (14.3) | 0.702 |  | 4 (10.5) | 1 (8.3) | ＞0.999 |
| Anastomotic leak | 7 (14.0) |  | 4 (13.8) | 3 (14.3) | ＞0.999 |  | 5 (13.2) | 2 (16.7) | ＞0.999 |
| Hemorrhage | 4 (8.0) |  | 3 (10.3) | 1 (4.8) | 0.849 |  | 3 (7.9) | 1 (8.3) | ＞0.999^‡^ |

AEs, adverse effects; NCRT, neoadjuvant chemoradiotherapy; PD, progressive disease; PR, partial response; SD, stable disease; TRG, tumor regression grade.

*Statistically significant values are given in bold. ^†^Mann-Whitney *U* test. ^‡^Fisher's exact test. *χ*^2^ test was used unless otherwise specified.
